# Overcrowding and Consumption

**Published:** 1901-02-02

**Authors:** 


### OVERCROWDING AND CONSUMPTION.

Among the many points upon which light is thrown by the interesting Annual Report issued by Mr. Shirley Murphy, medical officer to the County of London, one of the most important is that of the relation between mortality, and more especially mortality from phthisis and overcrowding. By a series of diagrams he shows in the first instance how steadily the mortality rises in proportion to the degree in which overcrowding prevails. Sorting out the sanitary districts into groups, according to the amount of overcrowding met with in each and taking as a datum line the death rate occurring in the least over-crowded group of districts, it is shown that, step by step, almost exactly in proportion as the amount of overcrowding increases the death rate rises. Then, dealing in the same way with the phthisis death rate alone, he shows that, not only does the same thing occur, but that the rise in the phthisis death rate which accompanies increased degrees of overcrowding is far greater than the rise in the general death rate taking place in the same circumstances. Even subtracting all the deaths from phthisis, there is a striking rise in the death rate in proportion as the overcrowding increases. But this rise is nothing to what occurs in regard to phthisis, for it is shown that with the higher degrees of overcrowding the accompanying increase in the death rate from phthisis is proportionately more than twice as great as that in the death rate from all other causes. It is true that phthisis is most common among the poor; it is true also that phthisis leads to poverty; and again that, however poverty arises, overcrowding is its common and almost necessary accompaniment. Thus the problem is a somewhat complicated one. Nevertheless the disproportionate degree to which the death rate from phthisis is raised among those who pass their lives in overcrowded dwellings is so striking that it seems impossible to avoid the conclusion that if we could but lessen the overcrowding we should cut at the root of one of the most potent causes of consumption. Whatever may be the intricacy of the problem, we cannot permit ourselves to be content with a state of affairs which results in the phthisis death rate in some districts being more than four times as high (per 1,000 living) as it is in other more favoured portions of the Metropolis. It must be remembered that even the contrast between the different groups of districts set out in Mr. Shirley Murphy's report, striking as it is, does not express the whole truth. The death-rate of each district is but an average. In some parts of each both the death rate and the overcrowding rate must be higher, and in others lower than the rest. Thus in those districts, such as Hampstead, in which the phthisis mortality is the lowest no doubt there are areas in which it is far lower than the figures tell us; while in Southwark and St. Luke, where it is the highest, one may be perfectly certain that the average phthisis mortality for the whole district, high as it is, does not in any way express the decimation of the population which is going on in some of the most densely populated areas. We may express the belief that if in the collection of statistics in regard to consumption it were possible to group together, not whole sanitary districts, but separate streets, classified according to overcrowding, much as was done in Mr. Charles Booth's great work in accordance with social condition, we should find revealed a state of affairs which would force the hands of our reformers and drive them to adopt measures to open out this great city and spread it over a far wider area than it covers at the present time.

